# Qualitative Attributes of Commercial Pig Meat from an Italian Native Breed: The Nero d’Abruzzo

**DOI:** 10.3390/foods11091297

**Published:** 2022-04-29

**Authors:** Andrea Ianni, Francesca Bennato, Camillo Martino, Maurizio Odoardi, Agostino Sacchetti, Giuseppe Martino

**Affiliations:** 1 Faculty of Bioscience and Technology for Food, Agriculture and Environment, University of Teramo, 64100 Teramo, TE, Italy; aianni@unite.it (A.I.); fbennato@unite.it (F.B.); 2 Department of Veterinary Medicine, University of Perugia, 06126 Perugia, PE, Italy; camillo.martino@studenti.unipg.it; 3 Dipartimento Agricoltura della Regione Abruzzo, Via Catullo, 65100 Pescara, PE, Italy; maurizio.odoardi@regione.abruzzo.it (M.O.); agostino.sacchetti@regione.abruzzo.it (A.S.)

**Keywords:** biodiversity, meat quality, Nero d’Abruzzo pig, oxidative stability, volatile compounds

## Abstract

The main objective of this study was to characterize the main qualitative properties of commercial meat obtained from the Nero d’Abruzzo pig, a native breed of Central Italy. In order to valorize this animal production, a direct comparison was made with commercial meat products obtained from hybrid pigs. Over a period of 30 days, 76 steaks for each breed were purchased from the market, and samples were analyzed for total lipid content, fatty acids profile, Coenzyme Q10 content, resistance of meat to oxidative processes, volatile profile of cooked meat and electrophoretic profile of myofibrillar and sarcoplasmic proteins. Results showed the Nero d’Abruzzo to be richer in fat, which, however, is characterized by a higher concentration of α-linolenic acid, to which are attributed important health benefits. The native breed was also richer in Coenzyme Q10, a compound credited with antioxidant potential, whose presence could explain the better oxidative stability of meat samples that were cooked and stored for up to 7 days at +4 °C. In support of this last data, our finding of the characterization of the volatile profile of cooked meat, at the end of the storage period, showed in Nero d’Abruzzo a reduction in the accumulation of hexanal, notoriously associated with oxidative events and the development of unpleasant aromatic notes. In conclusion, aspects that can justify the nutritional superiority of this niche production compared to meat coming from cosmopolitan breeds have been identified.

## 1. Introduction

Starting from the mid-twentieth century, the livestock sector was characterized by a process of selection of commercial hybrid breeds characterized by high production standards, especially from a quantitative point of view, in order to adequately respond to the increase in the consumers’ demand for products of animal origin [1]. This phenomenon has therefore led to an impoverishment of genetic resources which, in the past had a fair distribution, especially in the mountainous and marginal areas further away from the most important urban centers [2]. Currently, especially in western countries, the recovery and preservation of biodiversity represent a pivotal issue in the zootechnical world and it concerns both the sphere of ruminants and that of monogastrics [3,4,5]. This aspect also concerns the pig sector, despite the fact that the consumption of this type of meat is constantly growing and therefore it is still strongly necessary to resort to the breeding of commercial hybrids with intensive management [6]. In this regard, it is worth mentioning that pork production accounts globally for 33% of total meat consumption, with large differences in consumption levels between different continents [7]. In Europe, meat consumption plays a pivotal role and specifically in Italy, has been recently estimated an annual per capita consumption of 55 kg, with pig meat contributing about 46%, followed by poultry and cattle meat (27% and 25%, respectively) [8]. As reported by Lebret and Čandek-Potokar [9], pork quality can involve several aspects, not only related to sensorial, nutritional and technological properties but also to the social sphere. This latter factor therefore includes cultural, ethical (animal welfare) and environmental aspects related to pork production, all of which are able to influence consumers’ perceptions of pig meat. Is therefore part of this context, the interest in valorizing the indigenous animal breeds of zootechnical interest.

**Figure 1:**
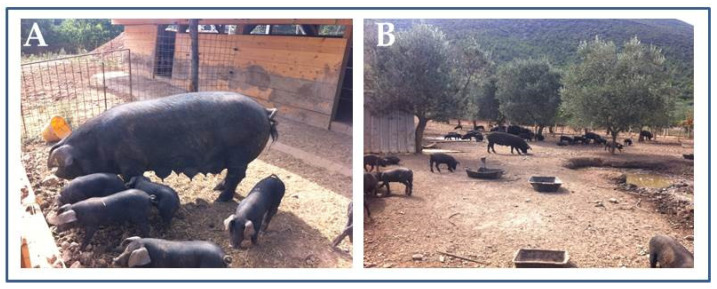
The Nero d’Abruzzo pig is an autochthonous breed from the Abruzzi region, in the center-south of Italy, characterized by dark color with straight frontal-nasal profile and ears turned forward and down to cover the eyes (**A**). These animals are also characterized by high rusticity and are commonly reared outdoors in disadvantaged areas (**B**).

The Nero d’Abruzzo pig is an autochthonous breed from the Abruzzi region, in the center-south of Italy, characterized by medium size and bristly dark hair, with a straight frontal–nasal profile and ears turned forward and down to cover the eyes (Figure 1A). These animals are also characterized by high rusticity and are commonly reared outdoors in disadvantaged areas (Figure 1B) for the production of fresh meat and traditional seasoned salami. This animal has not yet been the subject of genomic evaluations; however, it shares phenotypic traits with other native breeds located in Italy. From this point of view, is significant the study conducted by Davoli et al. [10] in which was investigated the genetic diversity among eleven pig breeds by typing SNPs in 23 coding genes. Among these breeds, five referred to Italian native breeds (Calabrese, Casertana, Cinta Senese, Mora Romagnola and Nero Siciliano). The result of this study showed for these breeds a good level of genetic differentiation; it is therefore plausible that the interaction with different environments has somehow had effects on the genome of phenotypically similar animals, and this could also characterize the Nero d’Abruzzo in comparison with other breeds.

The Nero d’Abruzzo experienced a condition of demographic decline until a few years ago, after which conservation programs were developed in order to prevent the extinction of this native breed which currently accounts for a population size of about 100 sows. Animals are distributed evenly throughout the Abruzzi region in a number of small farms, where these pigs are raised more for traditional and social reasons rather than for strictly commercial purposes. The recovery and valorization of these genetic resources of zootechnical interest at risk of extinction pass through an activity of characterization of the productions deriving from these animals, highlighting those peculiarities that can induce the consumer to prefer these products over those normally available on the market. By way of example, it might be useful the study conducted by Madonia et al. [11], in which a qualitative comparison was made between salami made by using meat from Nero Siciliano pigs and white pigs. The study made it possible to attribute a significantly higher health value to the product obtained from the rustic breed in terms of atherogenic and thrombogenic indices, as a direct consequence of the characteristic composition of meat fatty acids. This study was aimed at filling the knowledge gaps regarding the quality of the meat obtained from the Nero d’Abruzzo pig, which has never been the subject of such evaluations. Therefore, was specifically performed a comparison of specific qualitative parameters between commercial fresh meat obtained from the Nero d’Abruzzo pigs and meat deriving from commercial hybrid pigs. For this reason, attention was specifically focused on what the market makes available to the final consumer, without wanting to enter into the merits of the zootechnical aspects that can influence these parameters.

## 2. Materials and Methods

This study was conducted on commercial meat samples directly purchased from retail distribution centers. The experimentation therefore did not include tests on animals or the introduction of breeding practices other than those normally adopted. For this reason, it was not necessary to request evaluation and approval by an ethics committee.

### 2.1. Experimental Design

The study provided for the recruitment and comparison from a nutritional point of view of commercial fresh meat obtained from the Nero d’Abruzzo (NA) pigs and commercial hybrid (CH) pigs. Meat was specifically collected from different areas of commercial distribution located in the Abruzzi region.

For each type of animal were collected 76 samples of fresh meat (all coming from different animals with an age of 7–8 months; gender ratio 50:50) were packed in polystyrene trays and covered with a plastic film; all samples have been recruited over a period of 30 days and came from different animals but from the same anatomical cut, corresponding to the *Longissimus dorsi* between the 10th and 14th rib. All portions of meat were taken on the day of packaging, which occurred approximately 24 h after the slaughtering. Specifically, reference was made to the overwrap packaging in order to standardize the meat sampling in each of the distribution centers involved. Then, all the samples reached the laboratory at a controlled temperature of 4–5 °C and within 90 min of collection.

Meat was then subjected to the following analysis: evaluation of the total lipid content, characterization of the fatty acid profile, determination of the oxidative profile on raw meat and after cooking and storage at +4 °C for up to 7 days, dosage of coenzyme Q_10_ and identification of volatile compounds generated as a consequence of meat cooking. Furthermore, a basic proteomics investigation was performed aimed at defining the electrophoretic profile of myofibrillar and sarcoplasmic proteins. With specific regard to the cooking of the meat, the following procedure was observed: samples of 75–80 g and about 2.5 cm of thickness were inserted in a plastic bag and cooked in a water bath (Grant Instruments Ltd., Barrington, UK) until upon reaching a core temperature of 70 °C (Minitherm HI8751 temperature meter and probe, Hanna Instruments Ltd., Bedfordshire, UK); samples were then tempered at room temperature and left to cool at 4 °C overnight.

The aliquots of meat not immediately analyzed upon arrival in the laboratory were vacuum packed and frozen at −20 °C waiting to be used.

### 2.2. Moisture, Total Intramuscular Fat, Fatty Acid Profile and Lipid Oxidation

Moisture was determined according to the official method of the Association of Official Analytical Chemists (AOAC, 1984) [12].

The quantitative analysis of total intramuscular fat was performed following the official procedure reported by Horwitz and Latimer [13].

The evaluation of the fatty acid profile in meat was instead performed by following the procedure previously reported by Ianni et al. [14] with slight modifications. Briefly, 4 g of each meat sample were previously minced and then homogenized with Ultra-Turrax T-25 in 75 mL of Folch solution (chloroform-methanol; 2:1 *v*/*v*). After stirring for 5 h at room temperature, samples were filtered overnight in presence of sodium chloride. The chloroform phase was then recovered and the solvent was evaporated to dryness at 38 °C with a Strike-Rotating Evaporator (Steroglass S.r.l., Perugia, Italy). Forty mg of fat were then weighed and mixed with 4 mL of hexane containing 80 µL of methanol and 80 µL of sodium methoxide in order to induce the formation of the fatty acid methyl esters (FAME). The FAME detection was performed by a gas chromatograph (Focus GC; Thermo Scientific, Waltham, MA, USA) that exploited hydrogen as carrier gas. The instrument was equipped with a capillary column (Restek Rt-2560 Column fused silica 100 m × 0.25 mm highly polar phase; Restek Corporation, Bellefonte, PA, USA) and a flame ionization detector (FID). The ChromeCard software (ThermoQuest Italia S.p.A., Rodano, MI, Italy) was used for the quantification of peak areas, and individual fatty acids (FA) were expressed as relative percentages of total FAMEs. The FA identification was achieved by comparing the retention time of the standard mixture (FIM-FAME7-Mix; Matreya LLC, State College, PA, USA) and the value associated with each FA was used for the calculation of total polyunsaturated fatty acid (PUFA), saturated fatty acids (SFA) and monounsaturated fatty acids (MUFA); through the formulas proposed by Ulbricht and Southgate [15] were also determined in meat the atherogenic index (AI) and the thrombogenic index (TI).

The lipid oxidation in meat samples was determined by the test of Thio-Barbituric Acid Reactive Substances (TBARS), following the protocol previously described [16]. Data were reported in µg equivalents of malondialdehyde per gram of sample (calculated on a dry matter basis).

### 2.3. Evaluation of Volatile Profile of Cooked Meat

The extraction and analysis of volatile compounds (VOC) in cooked pork were performed by using the protocol previously reported by Ianni et al. [14]. With specific regard to the adopted thermal program and the VOC identification was made reference to the protocol previously described [17]. The data associated with each VOC has been reported as a relative percentage with respect to the total identified compounds.

### 2.4. Determination of Coenzyme Q_10_

The Coenzyme Q_10_ (CoQ_10_), also called ubiquinone, was evaluated in samples of pork muscle tissue by exploiting the high-performance liquid chromatography (HPLC) and according to the procedure previously described by Martino et al. [18]. Briefly, the samples were subjected to saponification for 15 min at 80 °C in presence of a KOH solution. The extraction process was performed by exploiting hexane; after the solvent evaporation under a nitrogen stream, the extract was then resuspended in methanol and 20 µL of the obtained solution was injected into the liquid chromatograph system (Varian 9012, Walnut Creck, CA, USA). The HPLC determination was achieved by using an LC18 column (4.6 mm × 250 mm, 5 µm) and a UV detector (Varian 9050, Walnut Creck, CA, USA) set at a wavelength of 275 nm. The mobile phase was composed of HPLC grade 2-propanol and methanol and the CoQ_10_ determination was performed by referring to a calibration curve that was prepared by using a specific standard (Sigma Aldrich, Milan, Italy). Data were reported in µg/g of meat, on a dry matter basis.

### 2.5. Sodium Dodecyl Sulfate-Polyacrylamide Gel Electrophoresis (SDS-PAGE) of Sarcoplasmic and Myofibrillar Proteins

The extraction of sarcoplasmic and myofibrillar proteins from meat samples was achieved by following the procedure previously described by Joo et al. [19], and the protein concentration in the extracts was determined using the Bradford protein assay [20]. The Sodium Dodecyl Sulfate-Polyacrylamide Gel Electrophoresis (SDS-PAGE) was then used in order to obtain the protein separation based on their molecular weight. The analysis was performed using a 12% polyacrylamide running gel and a 6% polyacrylamide stacking gel. In each lane, volumes of samples corresponding to a total protein content of 15 µg were loaded and the electrophoretic run was performed at 150 V for 90 min at room temperature. The obtained gels were stained with Coomassie Brilliant Blue and destained in a solution composed of 40% methanol, 10% acetic acid and 50% distilled water (*v*/*v*/*v*). A wide range of prestained molecular weight markers (10, 15, 20, 25, 37, 50, 75, 100, 150 and 250 kDa; BioRad, Milan, Italy) were also loaded in the electrophoretic system in order to facilitate the assignment of the molecular weight to the displayed protein fractions.

### 2.6. Statistical Analysis

Statistical evaluations were performed by exploiting the SigmaPlot 12.0 Software (Systat software Inc., San Jose, CA, USA) for Windows operating system. The one-way ANOVA model was applied, and the post-hoc comparison was performed through Tukey’s test; statistical significance was attributed in presence of *p* values lower than 0.05 and 0.01.

## 3. Results

### 3.1. Total Lipids, Fatty Acids Profile and Lipid Oxidative Stability

The determination of the lipid content in meat samples showed a significant difference between the NA and CH pigs. Specifically, the NA showed higher fat content (3.51 ± 0.86% vs. 2.16 ± 0.55% for NA and CH respectively, *p* < 0.05); no differences were instead evidenced with regard to the meat moisture (*p* > 0.05).

The genetic aspect also seems to have influenced the quality of the fat present in the meat (Table 1). Specifically, the commercial NA meat was characterized by higher concentrations of palmitic acid (C16:0; 28.81 ± 1.75% vs. 27.32 ± 1.41% in NA and CH respectively, *p* < 0.05) and linolenic acid (C18:3 *cis*9, 12, 15; 0.92 ± 0.11% vs. 0.75 ± 0.09% in NA and CH respectively, *p* < 0.01), while higher values of stearic acid (C18:0) were detected in CH meat (13.78 ± 0.81% vs. 12.31 ± 0.77% in CH and NA respectively, *p* < 0.05). Finally, the significant difference between the sum of “other” fatty acids should be also reported, with a higher mean value in the CH samples (*p* < 0.05). However, these variations did not induce significant changes in the total sum of the individual fatty acid families (SFA, MUFA and PUFA; *p* > 0.05). Even for the atherogenic and thrombogenic indices, no significant variations were highlighted, although it should be emphasized that the greater value of TI for the CH samples showed a tendency close to the statistical significance (*p* = 0.068).

**Figure 2:**
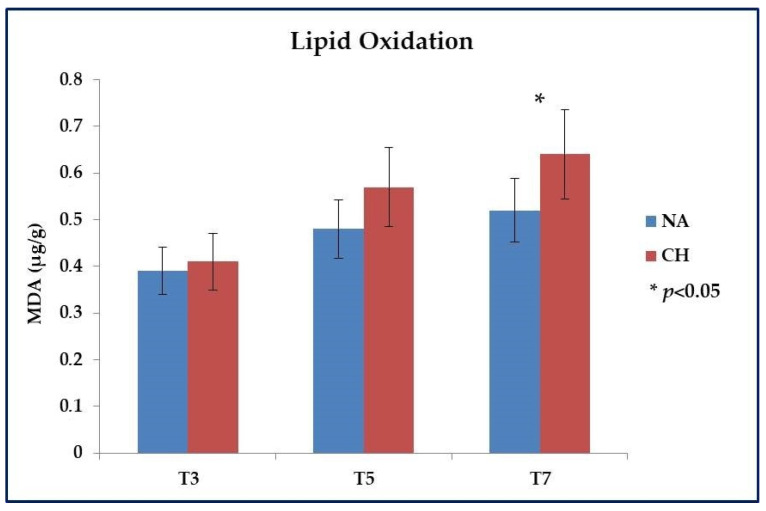
Lipid oxidation evaluated in meat obtained from the Nero d’Abruzzo pig (NA) and the commercial hybrid pig (CH) by using the test of Thio-Barbituric Acid Reactive Substances (TBARS). Analysis was performed on meat samples subjected to cooking and stored at +4 °C for up to 7 days (T7). Intermediate evaluations were performed 3 (T3) and 5 (T5) days after cooking. Data were reported in µg equivalents of malondialdehyde (MDA) per gram of meat; * *p* < 0.05.

Even for the lipid oxidative stability, a different profile was highlighted between the two types of meat. Specifically, the analysis was mainly focused on meat samples subjected to cooking and stored up to a maximum of 7 days at 4 °C, and the evaluation was based on the dosage of aldehydes that accumulated with the progression of the oxidative processes. As shown in Figure 2, within the first 3 days (T3) after cooking the oxidation level was almost identical among the samples under comparison (*p* > 0.05), but with the progress of time a different behavior emerged between the two types of meat, which led after 7 days (T7) to have a significantly lower level of lipid oxidation in NA samples (0.52 ± 0.05 µg MDA/g of meat vs. 0.64 ± 0.07 µg MDA/g of meat in NA and CH samples respectively; *p* < 0.05). It is important to underline that this investigation was conducted on a preliminary basis also on raw meat in order to verify that the starting values were similar and therefore that what was observed following cooking was solely the effect of the heat treatment. In this regard, comparable results were effectively observed between the two types of meat with values equal to 0.17 ± 0.03 µg MDA/g of meat for CH and 0.15 ± 0.02 µg MDA/g of meat for NA (*p* > 0.05).

### 3.2. Identification of Volatile Compounds (VOC) in Cooked Meat

The cooked meat deriving from the two pig breeds, NA and CH, was also subjected to the characterization of the volatile profile. This approach was conducted in order to deepen the evaluation of the oxidative processes and obtain information about the presence of compounds capable of influencing the meat flavor. The analysis was specifically performed on meat samples subjected to cooking and stored for 7 days at +4 °C.

As shown in Table 2, this approach allowed us to identify 17 VOCs, belonging to three different chemical families: aldehydes (10), ketones (1) and alcohols (6).

The only significant differences were found at the level of aldehydes, in which lower concentrations of hexanal and heptanal were observed in NA meat (*p* < 0.05 and *p* < 0.01 respectively), while 2-nonenal was characterized by lower concentrations in CH samples (*p* < 0.05).

### 3.3. Dosage of Coenzyme Q_10_ in Muscle Tissue

**Figure 3:**
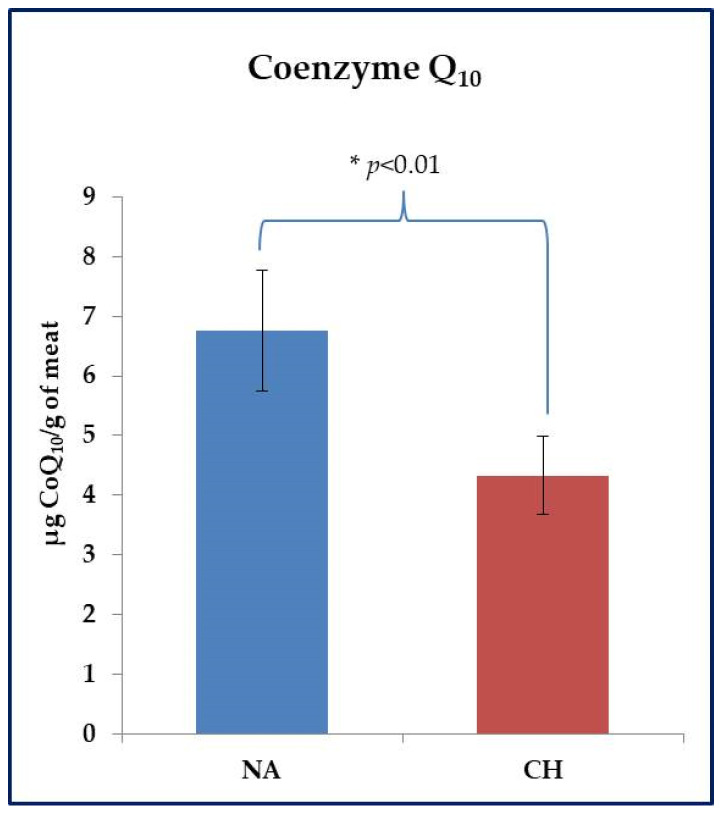
Evaluation of Coenzyme Q_10_ (CoQ_10_) in fresh meat obtained from the Nero d’Abruzzo pig (NA) and the commercial hybrid pig (CH) using an HPLC approach. Data were reported in µg of CoQ_10_ per gram of meat; * *p* < 0.05.

The CoQ_10_ assay was performed on fresh meat samples. With reference to this parameter, the animal breed also showed to be effective in inducing significant differences. As shown in Figure 3, the NA samples were found to be much richer in this compound than the CH ones (6.76 ± 0.71 µg CoQ_10_ /g of meat vs. 4.33 ± 0.45 µg CoQ_10_ /g of meat in NA and CH samples respectively, *p* < 0.01).

### 3.4. Characterization of Electrophoretic Profile of Sarcoplasmic and Myofibrillar Proteins

**Figure 4:**
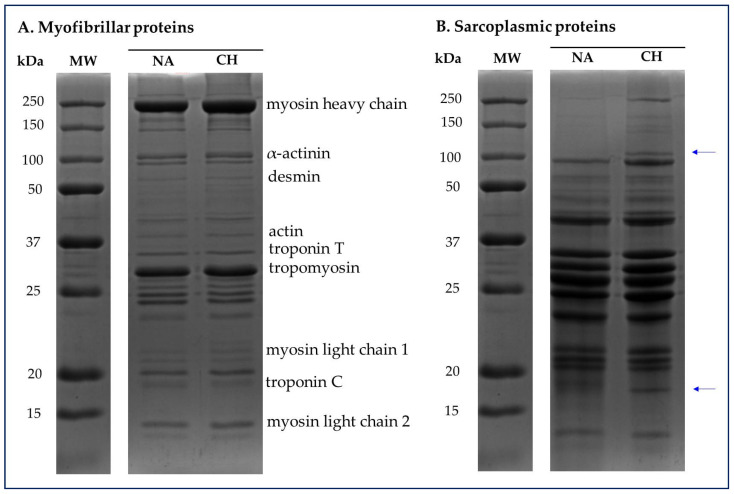
Representative electropherograms of sarcoplasmic and myofibrillar protein fractions detected in the muscle tissue of the Nero d’Abruzzo (NA) and commercial hybrid (CH) pigs. For both the myofibrillar (**A**) and sarcoplasmic (**B**) profiles, proteins in the molecular weight (MW) range between 250 and 10 kDa were visualized. The blue arrows highlight the bands detected only in CH samples and corresponding to an approximate molecular weight of 100–110 and 16–18 kDa respectively.

The SDS-PAGE was useful in highlighting the profiles of sarcoplasmic and myofibrillar proteins respectively present in the muscle tissue of the Nero d’Abruzzo and commercial hybrid pigs. In Figure 4 are shown representative electropherograms of the protein fractions in which it is possible to have a visual comparison between the samples obtained from NA and CH pigs. Specifically, for both the myofibrillar and sarcoplasmic profiles, proteins in the molecular weight range between 250 kDa and approximately 10 kDa were visualized.

With specific regard to myofibrillar proteins (Figure 4A) no differences were identified between the various samples used in the comparison, while in the case of the analysis of the sarcoplasmic profile, it should be emphasized that in all the CH samples were detected bands that are not present in the NA samples. These bands specifically correspond to proteins with an approximate molecular weight of 110 and 18 kDa.

## 4. Discussion

This study aimed to analyze and make a comparison between commercial pork meat obtained from an autochthonous breed and commercial hybrid pigs; this approach was intended to valorize a genetic resource of zootechnical interest not very widespread, highlighting the nutritional characteristics of potential interest for consumers. 

First of all, the genetic aspect showed to induce marked effects in the quantity of fat present at the intramuscular level, which was higher in the NA samples. The obtained results are almost comparable with those reported by other authors who compared the pork obtained from rustic breeds with that of commercial breeds [21,22]. This finding was largely justified by differences from the metabolic point of view and therefore by a greater lipid synthesis in rustic animals [23]. However, even the feeding protocol and the livestock production system could have influenced this parameter; it is in fact quite established that animals reared with free-range systems tend over time to accumulate greater quantities of fat than commercial hybrids reared with intensive systems, in response to the need to accumulate greater savings of energy in order to interact in the most suitable way with stimuli and stress of an environmental nature [24]. In support of this consideration, the study previously performed by Pugliese et al. [25] should be mentioned, in which was evidenced a marked adiposity in Nero Siciliano pigs reared outdoors. Despite the fact that consumers tend very often to prefer leaner meats, it should still be reported that the high content of intramuscular fat in rustic breeds represents one of the strengths of this meat, since this translates into an improvement of the eating quality, with a reduction in the shear force during chewing, favoring the separation of muscle fiber and improving the sensation of tenderness and juiciness [26].

Interesting differences were also highlighted by the analysis and characterization of individual fatty acids. This analysis first of all highlighted a higher concentration of palmitic acid (C16:0) in the NA samples. The type of fatty acids present in products of animal origin is generally influenced by the type of diet administered and this aspect should certainly not be excluded from the discussion, however, the fact that the rustic breed shows higher concentrations of this fatty acid could also suggest a difference at the level of the biosynthetic mechanisms. From this point of view, cannot be excluded the involvement of enzymatic mediators in fatty acids’ metabolism. Specifically, C16:0 represents the product of the enzymatic activity of the fatty acid synthase (FAS), an enzyme involved in the primordial processes for the de novo production of palmitate starting from acetyl-coA, malonyl-CoA, and NADPH [27]. The expression pattern of this specific enzyme in NA pigs has not been evaluated. However, studies that involved other indigenous pig breeds, showed for these animals an increased expression of both genes and transcription factors strictly associated with fatty acid metabolism in different tissues [22,28]. In addition to this, it should be also considered that CH samples were found to be richer in stearic acid (C18:0), therefore it could also be reasoned on the fact that the reduced presence of C16:0 in these samples is not so much due to a lower FAS activity but by greater use of C16:0 as substrate by elongase-6 (Elongase of long-chain fatty acids family 6, ELOVL6). This is a microsomal enzyme, ubiquitously found in almost every tissue in mammals, that catalyzes the chain elongation of palmitate to stearate, therefore influencing the tissue fatty acid composition [29].

The last aspect of considerable interest about the fatty acid profile concerns the greater presence in NA meat of linolenic acid (C18:3 cis9,12,15), an omega-3 with a high health value since its consumption is associated with a lower risk of encountering pathologies of the cardiovascular system [30]. In this regard it must be said that several studies over time have tried to manipulate the diet of animals of zootechnical interest to obtain animal products richer in fatty acids of this type [31,32], therefore this finding represents a leading element in affirming the qualitative superiority of meat from the rustic breed. This presumed greater health value of NA meat is, at least in part, confirmed by the thrombogenic index which is precisely higher in CH meat, although it should be pointed out that this difference does not reach the limit of significance (*p* = 0.068). In a previous study focused on the qualitative traits of meat obtained from the Nero Siciliano pig (native breed of southern Italy), the comparison with the white pig showed lower values of both the atherogenic and thrombogenic indices [11]. This aspect was attributed by the authors to a greater presence of oleic acid, which in our study did not show any differences between the types of meat analyzed. It is therefore plausible that these autochthonous breeds, while sharing important phenotypic and genotypic aspects, maintain peculiar characteristics that could depend—at least in part—on the exact geographical location and therefore on the relationship with the environment.

Meat obtained from rustic breed pigs also showed greater resistance to oxidative processes, that are commonly responsible for meat quality deterioration as a consequence of the accumulation of compounds potentially dangerous for consumers’ health. The better oxidative stability of meat obtained from pigs reared in free-range systems, has been already evidenced by Martino et al. [18], who partially justified this finding with a greater presence in the meat of CoQ_10_ which showed to be more represented even in the NA samples analyzed in this study. This is a lipophilic compound essential for the production of ATP through the mitochondrial electron transport chain [33]. However, what is important for our study is that this compound is also attributed to antioxidant properties; specifically, it was assumed that CoQ_10_ has the ability to act as a free radical scavenger in its reduced form [34]. This aspect is also relevant due to the fact that CoQ_10_ is resistant to thermal stresses and therefore resists cooking very well. This characteristic therefore explains the potential role of the compound as an antioxidant in a cooked food product, but it is also relevant for the consumer, as it means that the biological function of Q_10_ can reach the consumer, carrying out a bioactive action of absolute importance. In accordance with what was reported, the experimentation performed by Nevrkla et al. [35] should be mentioned, which showed lower values of malondialdehyde in meat samples obtained from native breed pigs compared to commercial breeds. This finding was attributed by the authors solely to the genetic aspect since the experimental design envisaged zeroing out all the variables of a zootechnical nature (diet administered, sex, age, breeding environment).

Furthermore, the better resistance to oxidative processes in meat could partly derive from the farming system. In fact, in extensive systems, the pigs’ diet is partially based on self-feeding as the animals tend to feed on the plant species existing in the breeding area. This obviously leads these animals to have a much more varied and, above all, potentially richer in natural bioactive compounds which are very often characterized by a marked antioxidant action [36]. It is therefore plausible that these compounds, or their metabolites, can be absorbed by the animal’s tissues which are therefore enriched with a sort of natural preservatives that improve the oxidative stability of the products.

The analysis of the volatile profile was very useful to better investigate the data concerning the greater resistance of NA samples to oxidative processes. In this regard, in the meat obtained from the rustic breed, was found a lower concentration of hexanal, an aldehyde that is notoriously generated following the spontaneous or enzymatic oxidation of saturated fatty acids [37]. Specifically, this compound represents the most prominent volatile aldehyde associated with the oxidation of linoleic acid [38], which however is present in our study in overlapping concentrations between NA and CH meat. It is therefore plausible that the lower presence of this compound in the rustic breed is attributable not to a reduced concentration of the substrate useful for oxidation, but more likely to reduced efficiency of the oxidative process, an aspect that is connected to what was previously discussed about the potential role of CoQ_10_ [18,34] and the probable intake of natural antioxidants [36] through the self-feeding which characterizes the extensive farming system.

This result acquires particular importance also for the fact that hexanal, if present in high concentrations, induces in food products particularly negative effects associated with the accumulation of off-flavors characterized by grass-like and rancid note. For this reason, hexanal probably represents the major indicator of flavor deterioration in meat and meat products [39]. It is plausible that this evidence also reflects a better profile from the sensorial point of view, although this aspect has not been addressed in this study.

In addition to the quantitative and qualitative analyzes carried out on the lipid component, this study also sought to perform preliminary evaluations on the protein component. From this point of view, the electrophoretic investigation of the myofribillar and sarcoplasmic proteins was very interesting, highlighting a picture that exposes itself to more specific and in-depth evaluations. With regard to the myofibrillar component, it is well-known that the functionality of these kinds of proteins exert a marked influence on the technological properties of processed muscle foods, with particular reference to texture, tenderness and moisture retention [40]. The sarcoplasmic proteins are instead soluble proteins coming from the sarcoplasm, to which above all belong myoglobin, several enzymes of the glycolytic pathway and globular proteins characterized by low molecular weight [41]. The denaturation process of this kind of protein was demonstrated to be effective in modifying different qualitative attributes in meat, such as water holding capacity and color [42].

In the present study, no significant variations were highlighted in the electrophoretic profile of the myofibrillar proteins, while an interesting finding was obtained from the analysis of the sarcoplasmic fraction. Specifically, in all meat samples coming from the rustic breed, was registered the absence of two protein bands, corresponding to the molecular weights close to 100–110 kDa and 16–18 kDa. According to the theoretical molecular weight, it could be isoforms of the muscle glycogen phosphorylase (100 kDa) and myoglobin (17 kDa) [43]; however, hypothesizing the nature of these proteins solely on the basis of the molecular weight could be complicated and speculative. In any case, is interesting that this basic proteomic approach allowed us to highlight a difference that could represent the starting point for the identification of specific protein markers whose presentation could confirm or not the origin of the product, with the prospect of being able to discriminate between products coming from commercial hybrids or the autochthonous breed.

## 5. Conclusions

The present study allowed us to identify interesting aspects associated with the quality of meat obtained from the Nero d’Abruzzo, a native pig breed in the central Italy. Particular attention was paid to the total fat content and the fatty acid profile, the CoQ_10_ content was then evaluated, information about the oxidative stability of the product was recorded both by means of a colorimetric assay and through the identification of volatile compounds deriving from lipolytic processes, and finally, an exploratory proteomic investigation was carried out on the protein profile present in the muscle tissue. 

For the first time have been highlighted aspects that could be useful for the valorization of this little-known and widespread genetic resource of zootechnical interest. Specifically, the Nero d’Abruzzo meat showed to be richer in α-linolenic acid, the presence of which in animal products is commonly associated with important health benefits for consumers. Furthermore, the same samples were even characterized by better resistance to the oxidative process, thus allowing the product to be stored for relatively long periods of time without altering its nutritional and sensory properties. This last aspect could be justified, at least partially, by the higher content of CoQ_10_ found in this type of meat, which notoriously represents an antioxidant agent. Finally, of considerable interest, there is also the finding concerning a different profile of sarcoplasmic proteins in the Nero d’Abruzzo in comparison to commercial hybrid pigs. This aspect could represent the starting point for identifying markers that can discriminate products from different breeds, stemming the problem of food fraud that increasingly characterizes the market for food products obtained from the rustic breeds.

## Figures and Tables

**Table 1:** Fatty acid composition of commercial fresh meat obtained from the Nero d’Abruzzo pigs (NA) and commercial hybrid pigs (CH).

Fatty Acids ^1^	NA	CH	*p*-Value
C14:0	1.59 ± 0.18	1.77 ± 0.22	ns
C16:0	28.81 ± 1.75	27.32 ± 1.41	*
C18:0	12.31 ± 0.77	13.78 ± 0.81	*
**SFA**	**42.71 ± 3.55**	**42.87 ± 3.27**	**ns**
C14:1	0.09 ± 0.02	0.06 ± 0.01	ns
C16:1	2.87 ± 0.33	2.91 ± 0.41	ns
C18:1 *cis*9	40.3 ± 2.91	39.56 ± 3.08	ns
C18:1 *cis*11	1.59 ± 0.18	1.70 ± 0.20	ns
**MUFA**	**44.85 ± 3.64**	**44.23 ± 3.76**	**ns**
C18:2 *cis*9, 12	9.02 ± 0.88	8.86 ± 0.91	ns
C18:3 *cis*9, 12, 15	0.92 ± 0.11	0.75 ± 0.09	**
**PUFA**	**9.94 ± 0.82**	**9.61 ± 0.93**	**ns**
Others ^2^	2.50 ± 0.24	3.29 ± 0.29	*
AI	0.64 ± 0.07	0.64 ± 0.08	ns
TI	1.43 ± 0.12	1.48 ± 0.11	ns

^1^ Data are reported as mean relative percentages of total FAMEs ± S.D.; SFA: saturated fatty acids; ^2^ Sum of other types of fatty acids not identified with absolute certainty, present in traces and homogeneously distributed along the entire chromatographic profile; MUFA: monounsaturated fatty acids; PUFA: polyunsaturated fatty acids; AI: Atherogenic index; TI: Thrombogenic index: * *p* < 0.05; ** *p* < 0.01; ns: not significant.

**Table 2:** Volatile compounds (VOC) identified in meat obtained from the Nero d’Abruzzo pigs (NA) and commercial hybrid pigs (CH). The analysis was specifically performed on meat samples subjected to cooking and stored for 7 days at +4 °C.

VOC ^1^	NA	CH	*p*
**Aldehydes**			
butanal 3-methyl	0.92 ± 0.11	0.81 ± 0.28	ns
butanal 2-methyl	0.09 ± 0.04	0.11 ± 0.02	ns
pentanal	2.68 ± 0.79	2.39 ± 0.44	ns
hexanal	72.27 ± 1.97	75.06 ± 2.21	*
heptanal	2.46 ± 0.22	3.62 ± 0.41	**
2-heptanal	1.04 ± 0.23	0.92 ± 0.11	ns
2-nonenal	0.21 ± 0.04	0.13 ± 0.03	**
2-decenal	0.55 ± 0.08	0.37 ± 0.06	ns
octanal	2.31 ± 0.38	1.61 ± 0.23	ns
nonanal	2.57 ± 0.16	2.31 ± 0.25	ns
**Ketones**			
3-hydroxy-2-butanone	2.10 ± 0.20	2.00 ± 0.19	ns
**Alcohols**			
1-butanol 3-methyl	0.65 ± 0.08	0.54 ± 0.07	ns
1-pentanol	0.61 ± 0.09	0.54 ± 0.06	ns
1-hexanol	2.98 ± 0.21	2.65 ± 0.31	ns
1-octyn-3-ol	0.73 ± 0.11	0.57 ± 0.08	ns
2-octen-1-ol	1.62 ± 0.22	1.47 ± 0.21	ns
1-octanol-2-butyl	6.21 ± 1.42	4.90 ± 1.63	ns

^1^ Data are reported as mean relative percentages of total volatile compounds (VOC) ± S.D.; * *p* < 0.05, ** *p* < 0.01, ns: not statistically significant (*p* > 0.05).

## Data Availability

All data are made available by the corresponding author following reasonable request.
